# Effective Removal of Tetracycline Antibiotics from Water using Hybrid Carbon Membranes

**DOI:** 10.1038/srep43717

**Published:** 2017-03-03

**Authors:** Ming-kai Liu, Ying-ya Liu, Dan-dan Bao, Gen Zhu, Guo-hai Yang, Jun-feng Geng, Hai-tao Li

**Affiliations:** 1 School of Chemistry and Chemical Engineering, Jiangsu Normal University, Xuzhou 221116, China; 2 Institute for Renewable Energy and Environmental Technologies, University of Bolton, Bolton BL3 5AB, U.K

## Abstract

Antibiotic residues in drinking water have become a global problem, especially in developing countries. However, effective purification of water contaminated by antibiotics remains a great challenge. Here, we investigated the removing of tetracycline by carbon nanomaterials with different structures and surface functionalities. The result shows that a membrane of thick graphene oxide (GO) and activated carbon (AC) with a thickness of 15 μm can effectively remove 98.9% of tetracycline hydrochloride (TCH) from water by vacuum filtration. Structural analysis indicated that the AC nanoparticles were uniformly inserted into the GO interstitial sites without any aggregations. Also, GO sheets were loosened by the encapsulated AC nanoparticles, leading to the formation of numerous tiny pores (3–10 nm) that acted as channels for fluid passage, whereas the carbons and chemical groups on the GO surface adsorbed TCH. GO/AC membrane exhibits the best adsorption efficiency among the investigated materials, including pure GO, AC, carbon nanotube (CNT), and CNT/AC and GO/CNT hybrids.

Industrialization in the modern world has brought about the development of new products but has likewise generated novel contaminants, which could profoundly harm our environment^1^,^2^. The crisis of freshwater in many developing countries has been further aggravated by pollutants from chemical and biological species, which have serious effects on human health^3^. Pharmaceutical antibiotics, one of the most heavily used classes of drugs in medical therapy and the farming industry, have frequently been detected in soil, surface water, ground water, and drinking water. Most antibiotics cannot be fully absorbed and metabolized by humans and animals^4^,^5^,^6^. Some of the antibiotics that are used excessively have low biodegradability and can potentially cause a variety of adverse effects including acute and chronic toxicity, disruption of aquatic photosynthetic organisms, impact on indigenous microbial populations, and damage to antibiotic-resistant genes in microorganisms^7^,^8^. Thus, the presence of antibiotic residues in water poses serious risks to human and ecological and is a major concern^9^.

The traditional water treatment systems used to remove microorganisms from water and wastewater are not completely effective at removing antibiotics^10^. Hence, the risks to human health and the environment as well as the associated financial losses have increased the urgency to develop effective and efficient technologies to thoroughly remove antibiotics from water, in particular from drinking water. As well, the development of carbon nanomaterials has stimulated a renewed interest in utilizing these materials in adsorption applications for water purification because carbon nanomaterials can meet the criteria required for a good adsorption process as they possess a high surface area, large pore volume, and strong physical and chemical interactions with pollutants. For example, due to their high adsorption capacity, activated carbons (ACs) have been widely used in adsorption treatments to remove pollutants^11^,^12^. Carbon nanotubes (CNTs) are also considered a promising adsorbent because of their high aspect ratio, large surface area and mesoporous volume^13^,^14^. Moreover, graphene and graphene oxide (GO) exhibit improved performances in removing organic dyes, pharmaceutical antibiotics, and aromatic compounds due to their large specific surface area and/or massive functional groups on their surface^15^,^16^,^17^. GO, which is structurally similar to graphene, possesses a large quantity of oxygen-containing epoxy, hydroxyl, and carboxyl groups, and the presence of these functional groups has rendered GO highly hydrophilic and thus suitable for use in aquatic and biological environments^18^,^19^.

Graphene and graphene-based carbon nanomaterials might be particularly suited for removing organic pollutants possessing aromatic rings in their molecular structures because of the strong “π-π” electron interactions that occur between graphene and the contaminants. However, like any nanoscale material, carbon nanomaterials are prone to aggregate due to their ultrahigh surface energy^20^,^21^,^22^. Therefore, the development of carbon nanomaterials with large surface areas and/or porous structures, but with significantly reduced tendencies to aggregate, is of great interest to the scientific community, especially for the purposes of contaminant removal. To this end, membrane separation appears to provide a rational option, and the technology has been used in the water purification, food processing, pharmaceutical, and chemical industries^23^,^24^,^25^.

Until now, the majority of the membranes made for molecular separation have been based on polymeric materials, which include polyimide, polysulfone, and cellulose^23^,^26^. However, the widespread application of polymeric membranes is severely limited by a number of drawbacks including poor tolerance to acidic or alkaline reagents, high temperatures, and organic solvents. In contrast, carbonic materials have demonstrated abilities adsorbing dyes, antibiotics, and heavy metal ions in a natural adsorption method, but the adsorption process usually takes hours to days to complete. Thus, the development of a thin and light-weight separation membrane with a dramatically reduced filtration time and enhanced adsorption capacity is highly desirable. Here, we report that with the assistance of homemade hybrid carbon membranes including GO/AC, GO/CNT, and CNT/AC, we achieved high adsorption capacities and high filtration efficiencies for the removal of tetracycline hydrochloride (TCH) antibiotics and dyes such as rhodamine B and methylene blue from water. Finally, the structures of these membranes were analyzed, and their adsorption performances were investigated.

## Results

**Figure 1:**
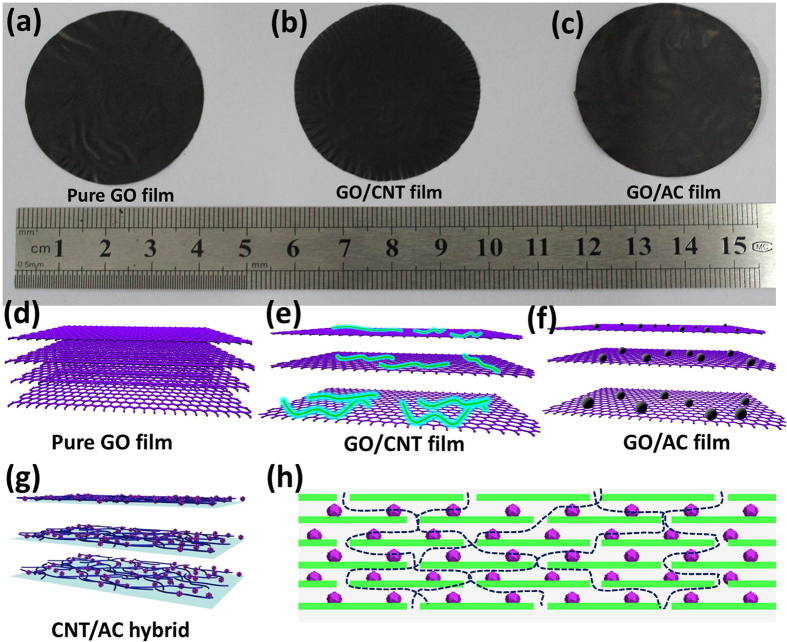
Optical images of (**a**) GO, (**b**) GO/CNT, and (**c**) GO/AC membranes. Schematic illustration of (**d**) pure GO, (**e**) GO/CNT, (**f**) GO/AC membranes and (**g**) the CNT/AC hybrid. (**h**) A possible route for the permeation of the TCH solution through contact with the GO/AC membrane.

Pure GO, CNT, and AC, and hybrid mixtures of GO/CNT, GO/AC, and CNT/AC were used to produce free-standing and flexible membranes based on an easy vacuum filtration method. The GO, GO/CNT, and GO/AC membrane morphologies are shown in Fig. 1a–c. These membranes resembled paper, possessed good mechanical flexibility (Figure S1a), and could be used directly in our purification experiments. Compared with GO/CNT and GO/AC, the membranes made of pure GO performed poorly due to its dense structure that arose from the strong π-π stacking^27^ (Fig. 1d). When CNTs and ACs were inserted into the interstitial sites of the GO nanoflakes, porous structures formed inside the hybrid membranes (as illustrated by Fig. 1e,f, also see the N_2_ gas adsorption result). However, the CNT/AC membranes exhibited poor mechanical strength and started to develop cracks and gaps only a few minutes after their formation (Figure S1b and S1c).

The potential water permeation route is presented in Fig. 1h. Due to the porous structures that emerged in GO following the introduction of CNTs and ACs, micro channels in the hybrids were created that accelerated the water flow through the membranes. Therefore, the suitability of the membranes for water purification purposes was enhanced by the hybridization approach and resulted in improved filtration efficiency and shorter filtration times.

**Figure 2:**
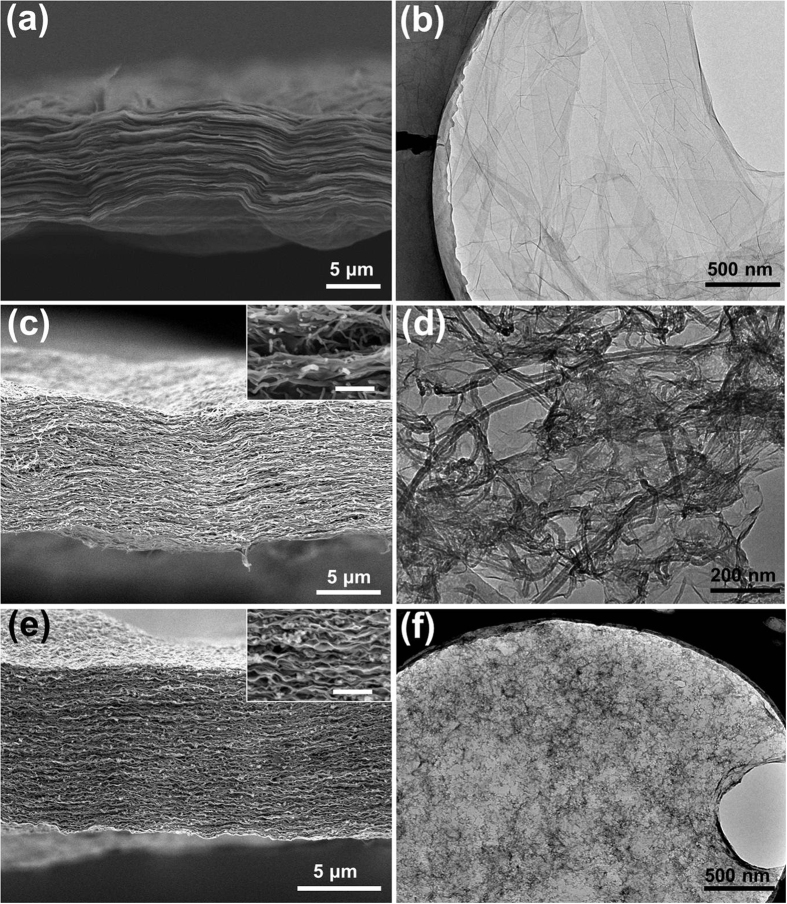
SEM images of a cross-section of (**a**) GO, (**c**) GO/CNT, and (**d**) GO/AC membranes, and TEM images of (**b**) GO, (**d**) GO/CNT, and (**f**) GO/AC membranes. The scale bar for the inset in (**c**,**e**) is 500 nm.

Scanning electron microscopy (SEM) images of cross-sections of the GO, GO/CNT, and GO/AC membranes are presented in Fig. 2a,c,e, respectively, with the transmission electron microscopy (TEM) images of the materials shown in Fig. 2b,d,f, respectively. The images show that the GO membrane had a dense structure and that the one-dimensional (1D) CNTs (Fig. 2d) were uniformly dispersed within the GO flakes, which was presumably assisted by van der Waals forces between them. As a result, the GO/CNT membrane exhibited a more porous structure compared with pure GO. The insert in Fig. 2c shows a SEM image of the GO/CNT membrane at a higher magnification, confirming the good co-dispersion between GO and CNTs. The GO/AC membrane showed a porous structure that was similar to that of GO/CNT, with the AC nanoparticles (Fig. 2e) homogeneously inserted into the interstitial sites of the GO flakes. The uniform dispersion is also seen in the TEM image (Fig. 2f). As well, no holes or cracks were observed in the GO/CNT and GO/AC membranes.

**Figure 3:**
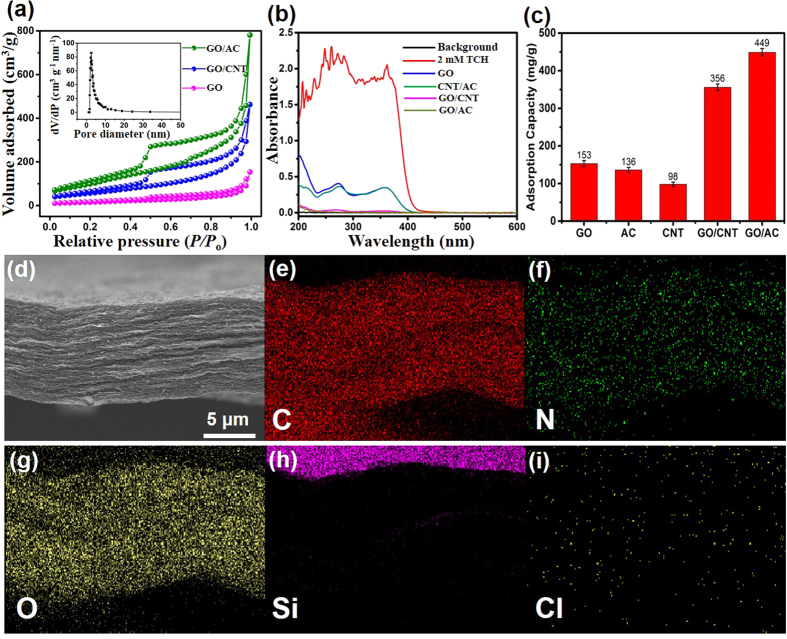
(**a**) N_2_ adsorption-desorption isotherms of GO/CNT, GO/AC, and pure GO membranes. The inset shows the pore size distribution of the GO/AC membrane. (**b**) UV-Vis absorption spectra of the initial TCH solution and the residue solutions obtained by filtration using GO, GO/CNT, and GO/AC membranes. (**c**) The saturated adsorption capacity of GO, AC, CNTs, GO/CNT, and GO/AC membranes. EDS images (**d**) of a cross section of the GO/AC membrane and the corresponding element distribution of (**e**) carbon, (**f**) nitrogen, (**g**) oxygen, (**h**) silica substrate, and (**i**) chlorine.

The results of the nitrogen (N_2_) adsorption-desorption isotherms of the GO, GO/CNT, and GO/AC membranes is shown in Fig. 3a. Both GO/CNT and GO/AC exhibited an apparently enhanced adsorption-desorption intensity compared with the pure GO membrane. The GO/CNT and GO/AC isotherms were categorized as type IV with hysteresis loops based on relative pressures (P/P_o_) between 0.4 and 1.0, which decisively confirmed their mesoporous features^10^. Also, the corresponding analysis of the GO/AC pore size distribution (inset in Fig. 3a) indicated that the pores were about 3–10 nm in size. Due to the excellent dispersion of the CNT and AC nanoparticles in GO, high specific surface areas of 414 m^2^/g for GO/AC and 326 m^2^/g for GO/CNT were achieved, which was much higher than the surface area of the pure GO membrane (~86 m^2^/g).

The UV-Vis absorption spectra of the initial TCH solution (20 mL, 1 mg/mL) and the TCH residue solutions following by filtration with GO, GO/CNT, CNT/AC, and GO/AC membranes are shown in Fig. 3b. It can be seen that the initial TCH solution had a strong absorption ability in the UV region (before 420 nm). After being filtered by GO membrane, the solution showed a greatly reduced absorption intensity in the same region. In comparison, more pronounced decreases in the absorption intensity were observed in the residue solutions filtered using the GO/CNT and GO/AC membranes, which confirmed the higher adsorption abilities of GO/CNT and GO/AC for TCH, with the GO/AC membrane exhibiting the best result.

A comparison of the adsorption capacities of the different carbon membranes for TCH is shown in Fig. 3c. Pure GO sheets (Figure S2) exhibited a saturated adsorption capacity of 153 mg/g, which was comparable to previous results obtained using graphene or GO-based active adsorption materials^27^,^28^. The AC and CNT membranes displayed lower capacities of 136 mg/g and 98 mg/g, respectively. However, the adsorption capacities of the GO/CNT and GO/AC hybrid membranes were 356 mg/g and 449 mg/g, respectively, which indicated the full utilization of their interfacial functional groups and their synergistic effect for TCH filtration. Also, rhodamine B and methylene blue were used as contaminants to test the removing ability of GO/AC membrane. As seen in Figure S3, good adsorption efficiencies of about 95% can be achieved due to the high removing ability of GO/AC membrane.

We also conducted an energy-dispersive spectroscopy (EDS) test on a post-filtered GO/AC membrane to study the adsorbed TCH on the membrane. The EDS images of the samples that were tested are shown in Fig. 3c–i. The strong carbon element shadow resulted from the GO and AC matrices, whereas the oxygen element mainly came from the chemical groups of GO (also see Figure S4). Uniformly distributed nitrogen and chlorine elements were likewise detected, confirming that the TCH molecules were indeed trapped by the membrane. Fourier transform infrared (FTIR) spectra of the GO, GO/CNT, and GO/AC membranes are shown in Figure S4. As seen in the spectra, GO exhibited a strong oxygen-containing characteristic from functional groups such as C = O in the carboxyl group (1722 cm^−1^), C-O from the epoxy group (1231 cm^−1^), C-O in the alkoxy group (1043 cm^−1^), and O-H stretching vibrations (3400 cm^−1^)^29^,^30^,^31^. As well, Figure S4 indicates that the mixing of CNTs and ACs with GO did not reduce the IR absorption strength of the GO oxygen-containing groups, indicating that the hybridization did not have any harmful effects on these groups.

**Figure 4:**
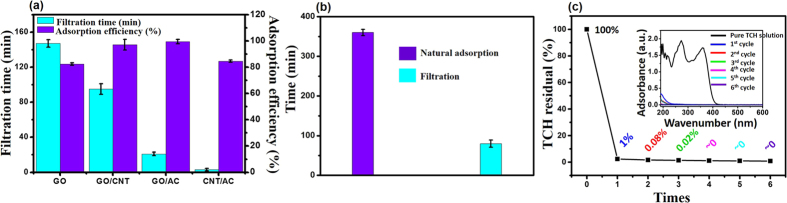
(**a**) The adsorption efficiencies and filtration times of the TCH solution (0.4 mg/mL, 20 mL) using pure GO, GO/CNT, GO/AC, and CNT/AC membranes. (**b**) Comparison of the separation time required for the natural adsorption method and the membrane filtration using GO/AC membrane. (**c**) The residual percentage of TCH in the solution after each filtration. UV-Vis spectra of TCH solutions filtered at different cycles using the GO/AC membrane (insert).

The adsorption capacity can be converted into an adsorption percentage, which was 82.3%, 96.1%, 98.9%, and 84.5% for the GO, GO/CNT, GO/AC, and CNT/AC membranes, respectively, following once filtration through each membrane (Fig. 4a). In addition, the filtration time (with 0.4 mg/mL TCH solution, 20 mL) was markedly reduced from 144 min with pure the pure GO membrane to 74 min with the GO/CNT membrane and to only 19 min with the GO/AC membrane (the filtration time was only 5 min for CNT/AC membrane, but this very short duration was likely caused by the cracks and holes in the membrane). Clearly, the GO/AC membrane demonstrated the best results in terms of both high adsorption capability and short filtration time.

In order to compare our membrane method with natural adsorption, we carried out a test of the two methods under identical conditions. Using a natural GO/AC mixture and the GO/AC membrane separately, we measured the adsorption of TCH in solution (0.4 mg/mL, 80 mL) in order to achieve the target 95% rate of adsorption. In the test, a mixture containing an equivalent amount of GO and AC with the GO/AC membrane was dispersed into the solution to examine the natural adsorption process. Figure 4b shows that the natural adsorption method required 360 min whereas only 80 min were required for membrane filtration, which confirmed the higher separation efficiency using the GO/AC membrane. In addition, the TCH solution was repeatedly filtered using the GO/AC membrane to record the percentage of the TCH residue in the water after each filtration (Fig. 4c). After four filtration cycles, the TCH residue in the water was nearly zero (Fig. 4c insert), which once again confirmed the high capability of the membrane to remove TCH from the water.

**Figure 5:**
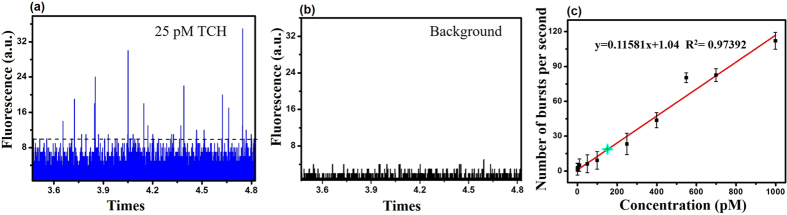
(**a**) Typical single molecule bursts of TCH at a concentration of 25 pM. (**b**) Fluorescence background of pure water. (**c**) The number of single molecular burst signals as a function of TCH concentration (0.55 pM, 1 pM, 10 pM, 50 pM, 100 pM, 250 pM, 400 pM, 700 pM, and 1000 pM). The error bars indicate the standard deviation of three separate measurements for each concentration of TCH. The green star symbol indicates a 150 pM TCH residual.

We also conducted a single molecule fluorescence experiment to determine the limit of TCH residue remaining in the sample solution, the results of which are summarized in Fig. 5a–c. The number of bursts from a single TCH molecule passing through the 488 nm laser focus volume increased in a TCH concentration-dependent manner. As shown in Fig. 5c, a linear correlation between the number of bursts and the concentration was observed using concentrations that ranged between 0.55 pM and 1.0 nM for the standard curve. As well, the detection limit was determined to be 500 fM. The results confirmed that the adsorption capacity of the GO/AC membrane could be effectively measured up to 99.99% by single molecule fluorescence technology, especially when the concentration was below the nM level, which represents the level of antibiotic contamination typically found in drinking water in developing countries. Finally, an additional benefit of the proposed technique is that it does not require any pre-separation or pre-treatment for the determination of the TCH quantity.

## Discussion

The excellent adsorption abilities of the GO-based membranes in regards to TCH can be ascribed to the characteristic carbon nanostructure and the abundant functional chemical groups on their surface. As previously mentioned, GO contains a number of oxygen-containing groups on its surface. Tetracycline consists of four aromatic rings with various functional groups on each ring including phenol, alcohol, ketone, and amino groups, which are expected to strongly interact with the GO aromatic rings and functional groups by hydrogen bonding, π-π electron interactions, and electrostatic interactions. These chemical interactions, in addition to a physical adsorption effect, contribute to the overall adsorption ability of GO towards TCH and its effective removal of TCH from the water.

The mixing of CNTs or AC with GO greatly enhanced the adsorption/purification ability of GO. A likely explanation for this enhanced effect is the markedly increased surface areas in the GO/CNT and GO/AC hybrid membranes compared with pure GO, as demonstrated by the results of the N_2_ gas adsorption tests. Furthermore, the increased surface areas resulted from the natural tendency of CNTs and AC to disperse into GO because they are all carbons with highly similar structures. Moreover, the hybrids possessed numerous tiny pores in their structures that were formed when the CNT and AC nanoparticles were inserted into GO sheets. CNT and AC nanoparticles were found within the interstitial sites of the GO flakes, which prevented the tight aggregation of the 2D flakes. Instead, the flakes formed a homogeneous mixture with the 1D CNTs or AC, resulting in the formation of uniformly dispersed membranes. This effect undoubtedly increased the overall surface area of the materials and facilitated the possible contacts between the TCH molecules and the carbon films for adsorption. Moreover, the tiny 1–3 nm pores present in the membrane acted as channels for water flow during the filtration processes. Consequently, a greater adsorption/removal ability of the GO/CNT and GO/AC membranes towards TCH was observed, with GO/AC demonstrating the best result.

## Conclusion

In this study, pure and hybrid membranes composed of 2D GO sheets, 1D CNTs, and AC were explored for the purpose of removing TCH antibiotic residues from water. Among them, the hybrid and flexible GO/AC membrane demonstrated an exceedingly strong adsorption capability towards TCH molecules. The underlying mechanism of the observed performance is believed to arise primarily from the intrinsically good adsorption ability of GO and AC, as well as the excellent dispersion of AC nanoparticles within the GO sheets. As a consequence of the dispersion, the adsorption surface areas in the hybrid structure were largely increased. Thus, the adsorption performance was greatly enhanced compared with pure GO or AC. Moreover, the GO/AC membrane exhibited the shortest filtration time and the highest filtration efficiency among all of the membranes tested, which can be attributed to the very porous structure formed during the hybridization process. Chemically, we attribute the effective adsorption capability to the strong interactions between GO/AC and TCH including hydrogen bonding, π-π electron stacking, electrostatic interactions, and the van der Waals forces between the GO functional groups and TCH. In summary, all these factors contributed to the high adsorption capacity (449 mg/g), which was much higher than the adsorption capacity of the pure GO membrane (251 mg/g). In addition, the result of single molecule fluorescence experiment confirmed the efficient removal of TCH residues to levels below nM range in drinking water. Overall, our results suggest that the development of hybrid membranes composed of structurally diverse but complementary nanocarbons provides a simple yet powerful and scalable approach to the removal of antibiotic residues in water^32^,^33^.

## Methods

### Materials

Natural flake graphite approximately 100 μm in size was purchased from Qingdao Graphite LTD., Qingdao, China. A modified Hummers method was used to prepare 1–5 μm GO sheets that were approximately 1 nm thick^34^. Pure CNTs with an average diameter of 30–50 nm and approximate length of 15 μm were purchased from Beijing Tiannai Nanotechnology Co. AC that was an average size of 100 nm were obtained from Nanjing XFNANO Materials Tech Co. TCH powder (analytical standard) was purchased from Sigma-Aldrich. All of the other chemicals were of analytical grade and were used as received without any further purification. Ultraclean water from a Milli-pore system was used in all of the experiments.

### Preparation of GO/AC, GO/CNT, CNT/AC, and pure GO membranes

A dispersion of GO/AC with a 2:1 GO to AC mass ratio was created by mixing 2.5 mg of AC with 5 mL of a GO suspension (1 mg/mL) under ultra-strong stirring and sonication. The GO/AC membrane was prepared by vacuum-filtering the obtained dispersion onto a porous polyvinylidene fluoride film (Xinya Filter Membranes-organo System, Shanghai, China) with a pore size of 0.22 μm. A free-standing GO/AC membrane was then obtained by separating the resulting GO/AC film from the fluoride substrate. Similarly, GO/CNT, CNT/AC, and CNT/AC membranes were generated using the same approach and the mass ratio of 2:1. The pure GO membrane was prepared using the same method.

### Molecule separation using the carbon membranes

The filtration/separation experiments were carried out with vacuum filtration equipment (Shanghai Weikai instrument equipment Co., LTD, China). Briefly, 20 mL of the TCH solution (0.4 mg/mL) was poured into a container equipped with the carbon membranes and the permeate solution was collected in the bottom compartment of the filter. The rejection rates of the TCH molecules were then calculated based on concentrations of the molecule in the original and permeate solutions, according to the following equation:


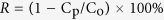


where C_o_ represents the concentration of TCH in the original solution and C_p_ is the concentration of TCH in the permeate solution. The concentration values in the permeate solutions were measured by UV-Vis spectroscopy. All of the data were calculated based on the results from at least three experiments.

### Characterization

Scanning electron microscopy (SEM) images were obtained using a Hitachi field-emission SEM (S4800, Japan) at 5 kV, and the corresponding energy-dispersive spectroscopy (EDS) was conducted using an Oxford X-Max^N^ 50 instrument (Oxford Instruments, UK). Transmission electron microscopy (TEM) images were obtained on a JEM 2100 instrument (JEOL, USA) operated at 200 kV. Fourier transform infrared (FTIR) analysis was performed on a Nicolet™ Nexus 470 FTIR spectrometer (Thermo Nicolet, USA) at a resolution of 4 cm^−1^. The UV-Vis spectra were recorded on a Lambda-25 spectrometer (Perkin-Elmer Inc. US). Brunauer-Emmett-Teller (BET) measurements were performed on an Autosorb-iQ-C analyzer (Quantachrome Instruments, USA) at 77 K.

### Single molecule fluorescence detection

The apparatus used for single molecule fluorescence detection was similar to a previously described apparatus^35^,^36^. Briefly, a 488-nm Gaussian beam was directed through a dichroic mirror and oil immersion objective (Apochromat 60×, NA 1.40, Nikon, Japan) and focused on a 5 μm area of a 0.2 mL sample solution supported on a cover glass. The size of the beam diameter on the back aperture of the objective was 3 mm. Fluorescence was collected using the same objective and imaged on a 50 μm pinhole to reduce out-of-focus fluorescence and other background noise. Blue fluorescence was filtered using long-pass and band-pass filters (XF3085 and 488/15 Notch filters, Omega Optical Filters, USA) before being focused onto an Avalanche Photodiode (APD). The dark count rate for the APD was below 100 counts per second. The output from the APD was coupled to a PC-implemented multichannel scalar card (MCS-Plus, EG&G, Canada). The power of the laser entering the microscope was adjusted using Neutral Density Filters (Thorlabs, UK) from 10 μW to 2 mW to perform the trapping experiments (70% of this incident power reached the sample). Time bins of 0.1–10 ms were used for the experiments, which were conducted at room temperature (∼20 °C) with the excitation laser power adjusted to 200 μW. A bin time of 1 ms was used and the total time for one experiment was 8 seconds^37^.

## Additional Information

**How to cite this article:** Liu, M.-K. *et al*. Effective Removal of Tetracycline Antibiotics from Water using Hybrid Carbon Membranes. *Sci. Rep.* **7**, 43717; doi: 10.1038/srep43717 (2017).

**Publisher's note:** Springer Nature remains neutral with regard to jurisdictional claims in published maps and institutional affiliations.

## Supplementary Material

Supplementary Information

## References

[b1] Huang H. . Ultrafast Viscous Water Flow through Nanostrand-Channelled Graphene Oxide Membranes. Nat. Commun. 4, 2979 (2013). 24352165 10.1038/ncomms3979

[b2] Sun H. . Three-Dimensional Superwetting Mesh Film Based on Graphene Assembly for Liquid Transportation and Selective Absorption. ChemSusChem. 6, 2377–2381 (2013). 23929792 10.1002/cssc.201300319

[b3] Rivera-Utrilla J., Prados-Joya G., Sánchez-Polo M., Ferro-García M. A. & Bautista-Toledo I. Removal of Nitroimidazole Antibiotics from Aqueous Solution by Adsorption/Bioadsorption on Activated Carbon. J. Hazard. Mater. 170, 298–305 (2009). 19464791 10.1016/j.jhazmat.2009.04.096

[b4] Watkinson A. J., Murby E. J., Kolpin D. W. & Costanzo S. D. The Occurrence of Antibiotics in an Urban Watershed: From Wastewater to Drinking Water. Sci. Total Environ. 407, 2711–2723 (2009). 19138787 10.1016/j.scitotenv.2008.11.059

[b5] Ji L., Chen W., Duan L. & Zhu D. Mechanisms for Strong Adsorption of Tetracycline to Carbon Nanotubes: A Comparative Study Using Activated Carbon and Graphite as Adsorbents. Environ. Sci. Technol. 43, 2322–2327 (2009). 19452881 10.1021/es803268b

[b6] Ji L. . Adsorption of Tetracycline on Single-walled and Multi-walled Carbon Nanotubes as Affected by Aqueous Solution Chemistry. Environ. Toxicol. Chem. 29, 2713–2719 (2010). 20836069 10.1002/etc.350

[b7] Hong P., Al-Jassim N., Ansari M. & Mackie R. Environmental and Public Health Implications of Water Reuse: Antibiotics, Antibiotic Resistant Bacteria, and Antibiotic Resistance Genes. Antibiotics 2, 367–399 (2013). 27029309 10.3390/antibiotics2030367 PMC4790270

[b8] Aristilde L., Marichal C., Miéhé-Brendlé J., Lanson B. & Charlet L. Interactions of Oxytetracycline with a Smectite Clay: A Spectroscopic Study with Molecular Simulations. Environ. Sci. Technol. 44, 7839–7845 (2010). 20866047 10.1021/es102136y

[b9] Hu W. . Graphene-Based Antibacterial Paper. ACS Nano 4, 4317–4323 (2010). 20593851 10.1021/nn101097v

[b10] Ai L. & Jiang J. Removal of Methylene Blue from Aqueous Solution with Self-Assembled Cylindrical Graphene–Carbon Nanotube Hybrid. Chem. Eng. J. 192, 156–163 (2012).

[b11] Kim S. H., Shon H. K. & Ngo H. H. Adsorption Characteristics of Antibiotics Trimethoprim On Powdered and Granular Activated Carbon. J. Ind. Eng. Chem. 16, 344–349 (2010).

[b12] Moussavi G., Alahabadi A., Yaghmaeian K. & Eskandari M. Preparation, Characterization and Adsorption Potential of the NH4Cl-induced Activated Carbon for the Removal of Amoxicillin Antibiotic from Water. Chem. Eng. J. 217, 119–128 (2013).

[b13] Yang W. . Adsorption Behavior and Mechanisms of Norfloxacin onto Porous Resins and Carbon Nanotube. Chem. Eng. J. 179, 112–118 (2012).

[b14] Ji L., Chen W., Zheng S., Xu Z. & Zhu D. Adsorption of Sulfonamide Antibiotics to Multiwalled Carbon Nanotubes. Langmuir 25, 11608–11613 (2009). 19725569 10.1021/la9015838

[b15] Gao Y. . Adsorption and Removal of Tetracycline Antibiotics from Aqueous Solution by Graphene Oxide. J. Colloid Inter. Sci. 368, 540–546 (2012). 10.1016/j.jcis.2011.11.015 22138269

[b16] Li W. . High-Density Three-Dimension Graphene Macroscopic Objects for High-Capacity Removal of Heavy Metal Ions. Sci. Rep. 3, 2125 (2013). 23821107 10.1038/srep02125 PMC3699809

[b17] Han Y., Xu Z. & Gao C. Ultrathin Graphene Nanofiltration Membrane for Water Purification. Adv. Funct. Mater. 23, 3693–3700 (2013).

[b18] Parrella A. . Acute and Chronic Toxicity of Six Anticancer Drugs on Rotifers and Crustaceans. Chemosphere. 115, 59–66 (2014). 24512989 10.1016/j.chemosphere.2014.01.013

[b19] Chowdhury I., Duch M. C., Mansukhani N. D., Hersam M. C. & Bouchard D. Colloidal Properties and Stability of Graphene Oxide Nanomaterials in the Aquatic Environment. Environ. Sci. Technol. 47, 6288–6296 (2013). 23668881 10.1021/es400483k

[b20] Gao S. J., Qin H., Liu P. & Jin J. SWCNT-intercalated GO Ultrathin Films for Ultrafast Separation of Molecules. J. Mater. Chem. A 3, 6649–6654 (2015).

[b21] Carabineiro S. A. C., Thavorn-amornsri T., Pereira M. F. R., Serp P. & Figueiredo J. L. Comparison Between Activated Carbon, Carbon Xerogel and Carbon Nanotubes for the Adsorption of the Antibiotic Ciprofloxacin. Catal. Today 186, 29–34 (2012).

[b22] Apul O. G., Wang Q., Zhou Y. & Karanfil T. Adsorption of Aromatic Organic Contaminants by Graphene Nanosheets: Comparison with Carbon Nanotubes and Activated Carbon. Water Res. 47, 1648–1654 (2013). 23313232 10.1016/j.watres.2012.12.031

[b23] Huang L., Zhang M., Li C. & Shi G. Graphene-Based Membranes for Molecular Separation. J. Phys. Chem. Lett. 6, 2806–2815 (2015). 26266866 10.1021/acs.jpclett.5b00914

[b24] Marchetti P., Jimenez Solomon M. F., Szekely G. & Livingston A. G. Molecular Separation with Organic Solvent Nanofiltration: A Critical Review. Chem. Rev. 114, 10735–10806 (2014). 25333504 10.1021/cr500006j

[b25] Shannon M. A. . Science and Technology for Water Purification in the Coming Decades. Nature 452, 301–310 (2008). 18354474 10.1038/nature06599

[b26] Pendergast M. M. & Hoek E. M. V. A Review of Water Treatment Membrane Nanotechnologies. Energy Environ. Sci. 4, 1946–1971 (2011).

[b27] Zhuang Y., Yu F., Ma J. & Chen J. Graphene as a Template and Structural Scaffold for the Synthesis of a 3D Porous Bio-Adsorbent to Remove Antibiotics From Water. RSC Adv. 5, 27964–27969 (2015).

[b28] Chen H., Gao B. & Li H. Removal of Sulfamethoxazole and Ciprofloxacin From Aqueous Solutions by Graphene Oxide. J. Hazard. Mater. 282, 201–207 (2015). 24755346 10.1016/j.jhazmat.2014.03.063

[b29] Yu F., Ma J. & Bi D. Enhanced Adsorptive Removal of Selected Pharmaceutical Antibiotics From Aqueous Solution by Activated Graphene. Environ. Sci. Pollut. R. 22, 4715–4724 (2015). 10.1007/s11356-014-3723-9 25331528

[b30] Zhang C., Ren L., Wang X. & Liu T. Graphene Oxide-Assisted Dispersion of Pristine Multiwalled Carbon Nanotubes in Aqueous Media. J. Phys. Chem. C 114, 11435–11440 (2010).

[b31] Lin Y., Xu S. & Li J. Fast and Highly Efficient Tetracyclines Removal From Environmental Waters by Graphene Oxide Functionalized Magnetic Particles. Chem. Eng. J. 225, 679–685 (2013).

[b32] Wang S., Sun H., Ang H. M. & Tadé M. O. Adsorptive Remediation of Environmental Pollutants Using Novel Graphene-Based Nanomaterials. Chem. Eng. J. 226, 336–347 (2013).

[b33] Ji L., Liu F., Xu Z., Zheng S. & Zhu D. Adsorption of Pharmaceutical Antibiotics on Template-Synthesized Ordered Micro- and Mesoporous Carbons. Environ. Sci. Technol. 44, 3116–3122 (2010). 20201519 10.1021/es903716s

[b34] Yang Z. . Photovoltaic Wire Derived From a Graphene Composite Fiber Achieving an 8.45% Energy Conversion Efficiency. Angew. Chem. Int. Ed. 52, 7545–7548 (2013). 10.1002/anie.201301776 23716484

[b35] Li H., Ying L., Green J. J., Balasubramanian S. & Klenerman D. Ultrasensitive Coincidence Fluorescence Detection of Single DNA Molecules. Anal. Chem. 75, 1664–1670 (2003). 12705600 10.1021/ac026367z

[b36] White S. S. . Characterization of a Single Molecule DNA Switch in Free Solution. J. Am. Chem. Soc. 128, 11423–11432 (2006). 16939265 10.1021/ja0614870

[b37] Li H., Zhou D., Browne H. & Klenerman D. Evidence for Resonance Optical Trapping of Individual Fluorophore-Labeled Antibodies Using Single Molecule Fluorescence Spectroscopy. J. Am. Chem. Soc. 128, 5711–5717 (2006). 16637638 10.1021/ja056997t

