# Supplementary material for: Effective Removal of Tetracycline Antibiotics from Water using Hybrid Carbon Membranes
Source: Sci Rep. 2017 Mar 3;7:43717. doi: 10.1038/srep43717 (PMC5334645; doi:10.1038/srep43717)
Supplement: Supplementary Information [file srep43717-s1.doc]

Electronic Supplementary Data

**Effective Removal of Tetracycline Antibiotics from Water using Hybrid Carbon Membranes**

Ming-kai Liu^a,#^, Ying-ya Liu^a,#^, Dan-dan Bao^a^, Gen Zhu^a^, Guo-hai Yang^a^, Jun-feng Geng^b*^, Hai-tao Li^a*^

School of Chemistry and Chemical Engineering, Jiangsu Normal University, Xuzhou, Jiangsu, 221116, China

^a^Contribution from the School of Chemistry and Chemical Engineering, Jiangsu Normal University, Xuzhou 221116, China

^b^Institute for Renewable Energy and Environmental Technologies, University of Bolton, Bolton BL3 5AB, U.K

*To whom correspondence should be addressed:

Hai-tao Li, School of Chemistry and Chemical Engineering, Jiangsu Normal University, Xuzhou China

E-mail: haitao@jsnu.edu.cn

Junfeng Geng, Institute of Renewable Energy and Environmental Technologies, University of Bolton, Bolton U.K

E-mail: J.Geng@bolton.ac.uk


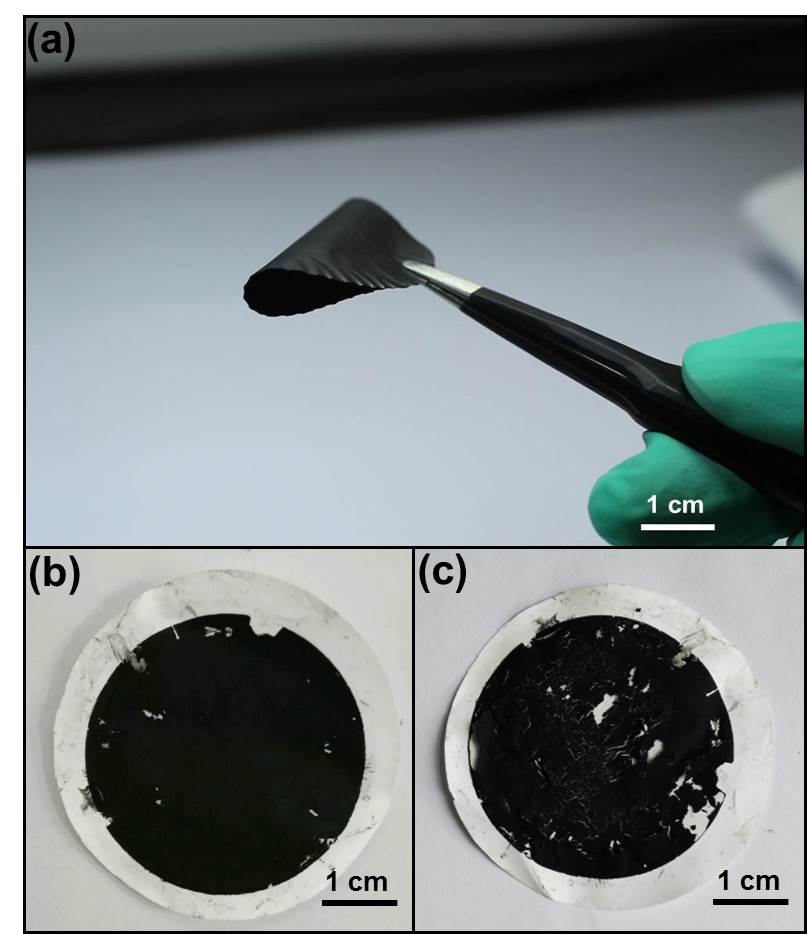


**Figure S1**. (a) Illustration of the flexibility of the GO/AC membranes. However, the CNT/AC membranes (b and c) exhibited numerous holes and cracks.


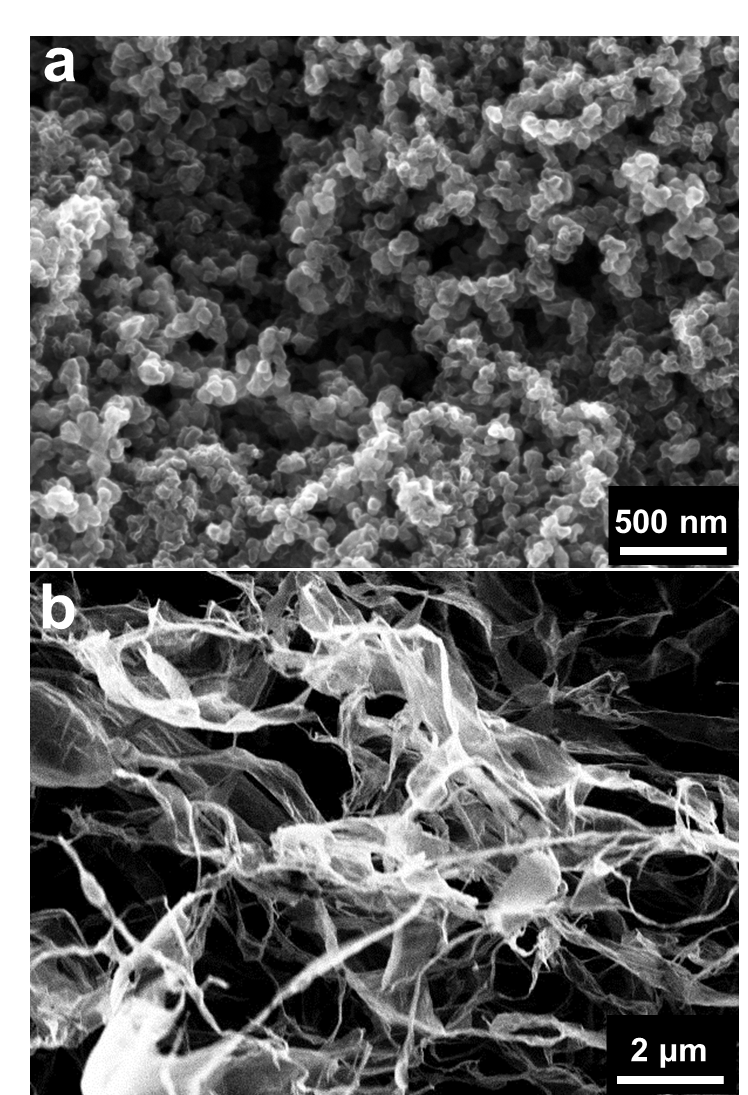


**Figure S2**. SEM images of CA particles and pure GO sheets.


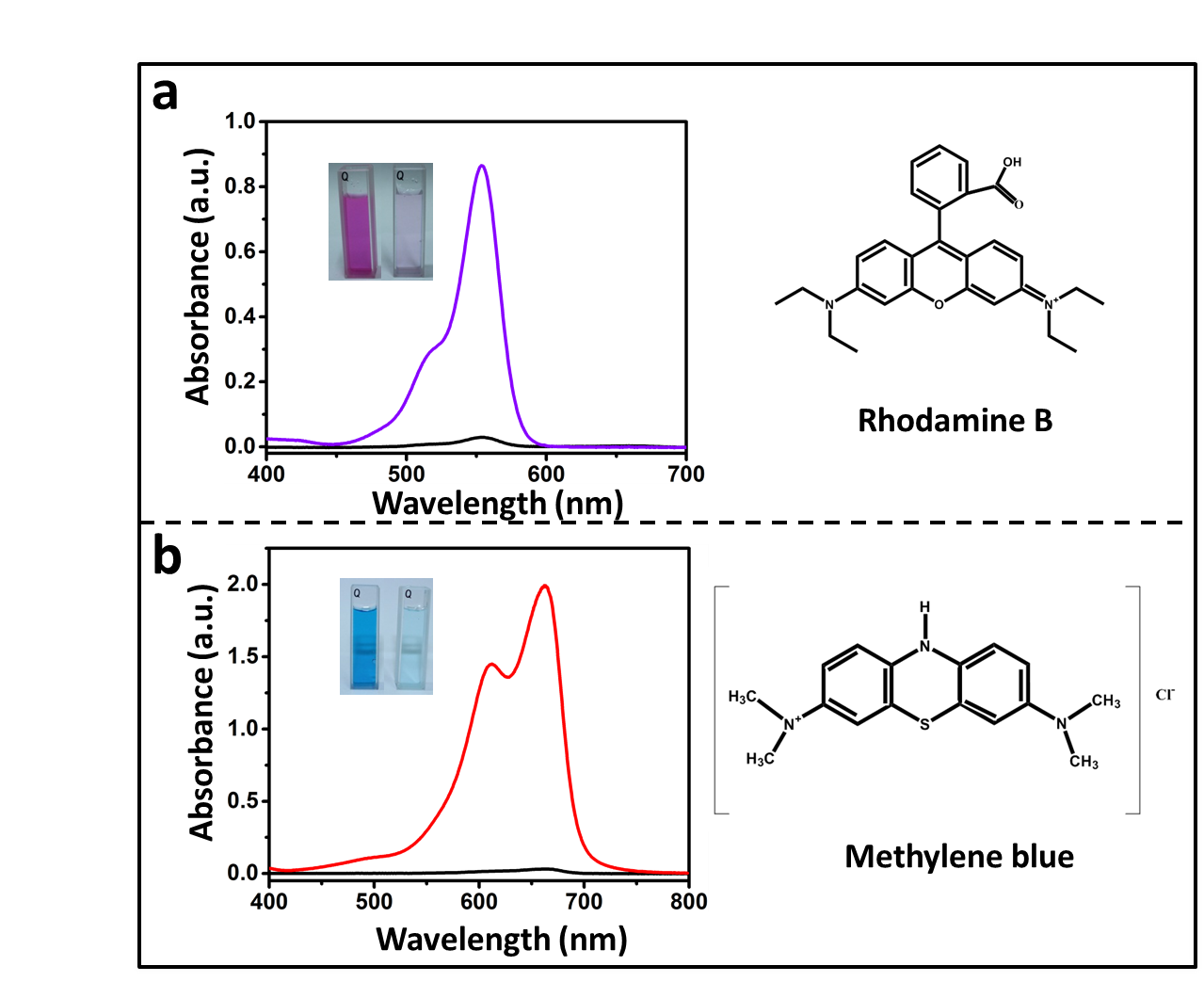


**Figure S3**. Uv-vis adsorption spectra of initial and residual Rhodamine B and Methylene blue filtrated by GO/AC membranes.


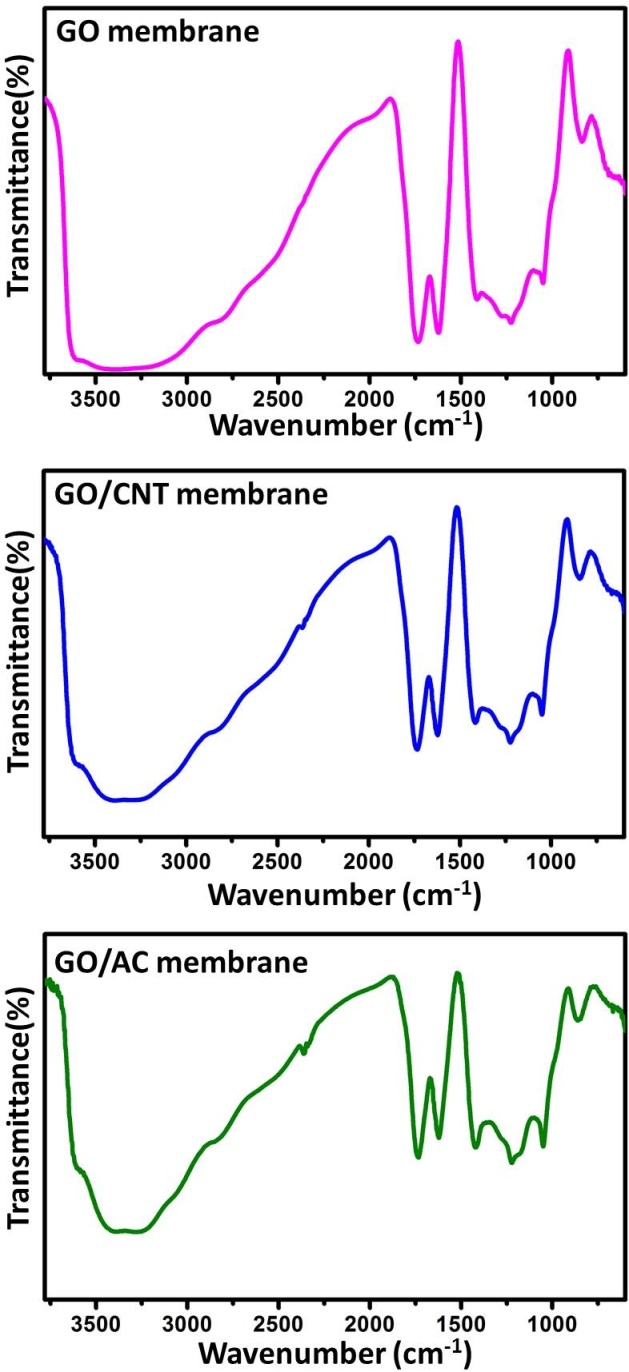


**Figure S4.** FTIR spectra of the pure GO, GO/CNT, and GO/AC membranes.
